# Governing glutaminolysis by regulation of glutaminase succinylation

**DOI:** 10.1007/s13238-021-00897-w

**Published:** 2021-12-16

**Authors:** Qingxia Ma, Hongfei Jiang, Leina Ma, Ying Meng, Dong Guo, Yingying Tong, Zhimin Lu

**Affiliations:** 1 The Affliated Hospital of Qingdao University, Qingdao Cancer Institute, Qingdao University, Qingdao, 266071 China; 2 Zhejiang Provincial Key Laboratory of Pancreatic Disease, The First Affiliated Hospital, and Institute of Translational Medicine, Zhejiang University School of Medicine, Zhejiang University, Hangzhou, 310029 China; 3 Cancer Center, Zhejiang University, Hangzhou, 310029 China; 4 Cancer Center, Beijing Luhe Hospital, Capital Medical University, Beijing, 101149 China

Of the 20 ribosomally coded amino acid residues, lysine (K) can be frequently and uniquely modified by many types of posttranslational acylation, including acetylation, succinylation, crotonylation, propionylation, butyrylation, glutarylation, malonylation, β-hydroxybutyrylation, and 2-hydroxyisobutyrylation in histones and other proteins (Li et al., 2018a; Xu et al., 2021). Among these acylations, succinylation is a commonly occurred modification, and histone H3K79 succinylation is identified in the transcription start sites of more than 7,000 genes, implying its critical role in the expression regulation of a large number of genes (Wang et al., 2017). Protein succinylation requires the high-energy metabolite, succinyl-coenzyme A (CoA), which is produced by several metabolic reactions mediated by different metabolic enzymes: (i) α-ketoglutarate (α-KG) dehydrogenase (α-KGDH) complex, which is composed of 2-oxoglutarate dehydrogenase, mitochondrial (OGDH), dihydrolipoamide succinyltransferase component of 2-oxoglutarate dehydrogenase complex (DLST) and dihydrolipoamide dehydrogenase (DLD) and catalyses the conversion of α-KG into succinyl-CoA; (ii) the succinyl-CoA synthetase (also known as succinyl-CoA ligase [SUCL]), which is a heterodimeric enzyme composed of an invariant α subunit encoded by *SUCLG1* and a substrate-specific β subunit encoded by either *SUCLA2* for ATP or *SUCLG2* for GTP production. SUCL catalyzes the reversible conversion of succinyl-CoA and ADP (or GDP) to CoA, succinate, and ATP (or GTP); (iii) 3-oxoacid CoA-transferase 1 (OXCT1, also known as succinyl-CoA-3-oxaloacid CoA transferase [SCOT]), catalyzing the reversible transfer of CoA from succinyl-CoA to acetoacetate, the first, rate-limiting step in ketolysis; (iv) mitochondrial enzymes through the catabolism of valine, isoleucine, methionine, thymine, and odd-number chain fatty acids or peroxisomal enzymes through the catabolism of long and very long fatty acids, 3-oxoadipate, or adipic acid via multiple routes (Chinopoulos, 2021).

Lysine acetyltransferase 2A (KAT2A, also known as GCN5), which is a histone acetyltransferase, was the first-identified protein succinyltransferase (Wang et al., 2017). In that report, we showed that KAT2A forms a complex with nucleus-translocated α-KGDH and utilizes α-KGDH-produced succinyl-CoA to succinylate histone H3 on K79 around the transcription start sites of genes (Wang et al., 2017). The co-crystal structures of the catalytic domain of KAT2A with succinyl-CoA reveal an octahedral complex composed of 24 KAT2A molecules, in which tyrosine 645 in the catalytic domain has an important role in the selective binding of succinyl-CoA over acetyl-CoA (Wang et al., 2017, 2018a). The supramolecular assemblies of KAT2A, high local concentration of succinyl-CoA generated by the KAT2A-associated α-KGDH complex, and high catalytic activity of KAT2A toward succinyl-CoA compensate for the relatively low nuclear concentration of succinyl-CoA to promote KAT2A-mediated histone succinylation (Wang et al., 2018a). In an *in vitro* setting, succinylation of histone H4 at K77 impacts nucleosome dynamics and promotes DNA unwrapping from the histone surface, thereby proteins such as transcription factors can rapidly access buried regions of the nucleosomal DNA (Jing et al., 2020). In human pancreatic ductal adenocarcinoma (PDAC) cells, highly expressed KAT2A regulates H3K79 succinylation in the promoter region of YWHAZ (encoding for 14-3-3ζ) to promote 14-3-3ζ expression, thereby preventing β-catenin degradation and subsequently increasing the expression of cyclin D1, c-Myc, GLUT1, and lactate dehydrogenase A (LDHA) to promotes glycolysis, cell proliferation, and migration and invasion of PDAC cells (Tong et al., 2020). These findings unveiled the instrumental mechanisms by which α-KGDH couples with succinyltransferase KAT2A to succinylate histone thereby regulating gene expression and promoting tumor development. However, as a critical oscillation process of the protein post-translational modification, whether protein desuccinylation is regulated in a local succinyl-CoA-dependent manner is largely unknown.

We recently reported that the kidney-type glutaminase (GLS) is succinylated in mitochondria. Importantly, the succinylation and desuccinylation of GLS is coupled-regulated by the association of GLS with SUCLA2, which is dynamically regulated in PDAC cells to promote

glutaminolysis for counteracting oxidative stress (Tong et al., 2021). Glutaminolysis is highly active in PDAC cells for anabolic processes (Son et al., 2013); stable isotope-assisted metabolomic analysis using [U-^13^C_5_] glutamine as the tracer showed that higher mass isotopologue distributions of glutamate and tricarboxylic acid (TCA) cycle metabolites were detected in PDAC cells than those in normal human pancreatic duct epithelial (HPDE) cells. Consistently, immunohistochemical (IHC) analyses of human PDAC specimens showed that GLS was overexpressed in PDAC compared to the normal adjacent counterpart tissues, and depletion or inhibition of GLS inhibited PDAC cell proliferation to a greater degree than inhibiting the proliferation of HPDE cells (Tong et al., 2021). These finding indicated that GLS promotes glutaminolysis in PDAC cells and tumor cell proliferation.

Notably, mass spectrometry analyses detected a previously unknow modification of GLS in PDAC cells, succinylation at K311, which occurred in a succinyl-CoA concentration-dependent manner. In addition, GLS K311 succinylation promoted GLS dimer and tetramer assembly by forming an interaction between succinylated K311 and H475 of an adjacent monomer in the interface, leading to enhanced activity of GLS. Reconstituted expression of GLS K311R in endogenous GLS-depleted PDAC cells decreased intracellular glutamate level, demonstrating an instrumental role of GLS K311 succinylation in glutamate production in PDAC cells (Tong et al., 2021).

Importantly, SUCLA2 was identified as a GLS-associated protein by mass spectrometry analyses, and this association was disrupted by oxidative stress, which enhanced GLS K311 succinylation. Treatment of PDAC cells with H_2_O_2_ resulted in the binding of p38 MAP kinase to SUCLA2. *In vitro* and *in vivo* experiments demonstrated that p38 directly binds to and phosphorylates SUCLA2 at serine (S)79. Mutation of S79 into alanine or treatment of the PDAC cells with a p38 inhibitor blocked oxidative stress-induced disassociation between SUCLA2 and GLS and diminished H_2_O_2_-induced GLS K311 succinylation. These findings suggested that alteration of the association between GLS and SUCLA2 modulates the available and local amount of SUCL-catalyzed succinyl-CoA thereby regulating GLS succinylation. Indeed, an *in vitro* experiment showed that purified SUCLA2 and SUCLG1 reduced succinyl-CoA-dependent GLS succinylation with correspondingly increased production of succinate, further supporting that SUCLA2-mediated conversion of succinyl-CoA to succinate reduces the available amount of succinyl-CoA for GLS succinylation.

**Figure 1:**
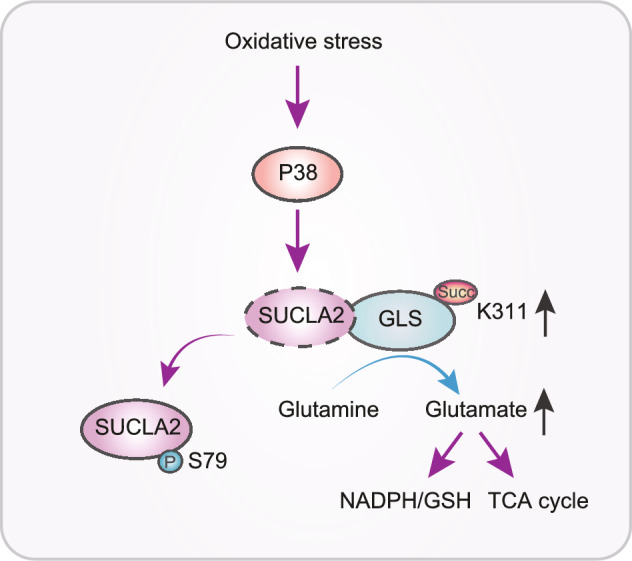
A schematic depicts the mechanism underlying SUCLA2-coupled regulation of GLS succinylation and activity in counteracting oxidative stress of tumor cells

As expected, expression of SUCLA2 S79A or GLS K311R reduced the fraction contribution of glutamine into TCA cycle in PDAC cells and decreased the cell proliferation rates. In addition, expression of these mutants reduced the ratios of glutathione (GSH)/oxidized glutathione (GSSG) and elevated the ratios of NADP^+^/NADPH and reactive oxygen species (ROS) levels and apoptosis rates of the PDAC cells in the presence and absence of H_2_O_2_ treatment. These results indicated that SUCLA2 S79 phosphorylation-promoted GLS K311 succinylation promotes glutaminolysis for cell proliferation and production of NADPH and GSH to maintain redox homeostasis thereby protecting PDAC cells from oxidative stress-induced apoptosis. Consistently, mouse studies showed that expression of SUCLA2 S79A or GLS K311R significant inhibited tumor growth and reduced the levels of GSH/GSSG ratios in tumor tissues (Fig. 1). Notably, the clinical relevance of GLS K311 succinylation was revealed by the IHC analyses of primary PDAC tissues showing that the mutually correlated levels of SUCLA2 pS79 and GLS K311 succinylation were substantially higher in the PDAC samples than those in the adjacent normal tissue samples and correlated with the advanced stages of PDAC and poor survival of the patients (Tong et al., 2021).

In addition to regulation of gene transcription by histone modification and mitochondrial functions, protein succinylation is also involved in modulation of other cellular activities. Mass spectrometric analysis identified tumor suppressor p53 succinylation at K120, which can be desuccinylatied by SIRT5. SIRT5 deficiency promotes p53 activity, target gene expression, and apoptosis in response to DNA damage (Liu et al., 2021). In addition, SIRT5 catalyzed K7 desuccinylation of mitochondrial antiviral signaling (MAVS) protein, resulting in reduction of aggregation and activity of MAVS after viral infection, the impairment of type I interferon production, and antiviral gene expression (Liu et al., 2020). These findings further underscore the multifaceted role of protein succinylation in critical cellular activities.

Metabolic enzymes can possess the moonlighting functions, which are distinct from their canonical functions and can directly participate in the posttranslational modifications of proteins, such as phosphorylation, acylation, methylation, hydroxylation, and O-GlcNAcylation (Lu, 2012; Li et al., 2016a, b, 2017, 2018a, b; Lu and Hunter, 2018; Wang et al., 2018b; Jiang et al., 2020, 2021; Xu et al., 2021). Nucleus-translocated α-KGDH couples with KAT2A acts as a writer for histone lysine succinylation whereas mitochondrial SUCLA2 decreases GLS succinylation by forming a complex with GLS and reducing available amount of succinyl-CoA for GLS succinylation. Importantly, succinylation of tumor-highly expressed GLS and glutaminolysis in PDAC are governed by dynamical association of GLS with SUCLA2, which confers tumor cells greater capacity to counteract oxidative stress and support tumor growth. Given the critical role of glutaminolysis in many types of cancer progression, the discovery of SUCLA2-coupled regulation of GLS succinylation in precise regulation of glutaminolysis provides GLS succinylation as a novel and promising diagnostic and therapeutic target for cancer care.
